# Eugenol alleviates transmissible gastroenteritis virus-induced intestinal epithelial injury by regulating NF-κB signaling pathway

**DOI:** 10.3389/fimmu.2022.921613

**Published:** 2022-08-16

**Authors:** Kang Wang, Daiwen Chen, Bing Yu, Jun He, Xiangbing Mao, Zhiqing Huang, Hui Yan, Aimin Wu, Yuheng Luo, Ping Zheng, Jie Yu, Junqiu Luo

**Affiliations:** ^1^ Institute of Animal Nutrition, Sichuan Agricultural University, Chengdu, China; ^2^ Key Laboratory of Animal Disease-resistant Nutrition, Sichuan Agricultural University, Chengdu, China

**Keywords:** transmissible gastroenteritis virus, eugenol, intestinal epithelial barrier, intestinal inflammation, immunity, weaned pigs

## Abstract

Increasing evidence supports the ability of eugenol to maintain intestinal barrier integrity and anti-inflammatory *in vitro* and *in vivo*; however, whether eugenol alleviates virus-mediated intestinal barrier damage and inflammation remains a mystery. Transmissible gastroenteritis virus (TGEV), a coronavirus, is one of the main causative agents of diarrhea in piglets and significantly impacts the global swine industry. Here, we found that eugenol could alleviate TGEV-induced intestinal functional impairment and inflammatory responses in piglets. Our results indicated that eugenol improved feed efficiency in TGEV-infected piglets. Eugenol not only increased serum immunoglobulin concentration (*IgG*) but also significantly decreased serum inflammatory cytokine concentration (*TNF-α*) in TGEV-infected piglets. In addition, eugenol also significantly decreased the expression of *NF-κB* mRNA and the phosphorylation level of *NF-κB P65* protein in the jejunum mucosa of TGEV-infected piglets. Eugenol increased villus height and the ratio of villus height to crypt depth in the jejunum and ileum, and decreased serum D-lactic acid levels. Importantly, eugenol increased tight junction protein (*ZO-1*) and mRNA expression levels of nutrient transporter-related genes (*GluT-2* and *CaT-1*) in the jejunum mucosa of TGEV-infected piglets. Meanwhile, compared with TGEV-infected IPEC-J2 cells, treatment with eugenol reduced the cell cytopathic effect, attenuated the inflammatory response. Interestingly, eugenol did not increase the expression of *ZO-1* and *Occludin* in IPEC-J2 cells. However, western blot and immunofluorescence results showed that eugenol restored TGEV-induced down-regulation of ZO-1 and Occludin, while BAY11-7082 (The NF-κB specific inhibitor) enhanced the regulatory ability of eugenol. Our findings demonstrated that eugenol attenuated TGEV-induced intestinal injury by increasing the expression of *ZO-1* and *Occludin*, which may be related to the inhibition of *NF-κB* signaling pathway. Eugenol may offer some therapeutic opportunities for coronavirus-related diseases.

## Introduction

Coronaviruses have strong variability and the ability to spread across species (1). Transmissible gastroenteritis virus (TGEV) is an enveloped, positive-sense, single-stranded RNA coronavirus with a length of about 28.5 kb, which is the main pathogen causing porcine gastroenteritis (2). Transmissible gastroenteritis (TGE) is a highly contagious enteric disease caused by TGEV, with clinical symptoms characterized by severe diarrhea, dehydration and vomiting in piglets (3, 4). The virus has now become widespread in several countries, causing severe economic losses to the swine industry (5). Therefore, it is necessary to understand the pathogenic mechanism of TGEV and find effective treatments.

The intestinal mucosal barrier includes epithelial cells and is the first line of defense against external pathogens from invading the intestine (6). It can rapidly activate early cellular responses and induce the production of various cytokines to act as a bridge between innate and adaptive immunity (7, 8). Therefore, the intact intestinal mucosal epithelium and its good physiological state are important guarantees for the healthy growth of animals. During viral infection, viruses alter or disrupt the normal architecture of the cellular intestinal barrier structure, further supporting viral entry, replication, and production of viral particles (9). Therefore, investigating the intestinal infection of piglets during TGEV infection has aroused considerable research interest.

Eugenol (C_10_H_12_O_2_), a well-known natural product with immunomodulatory and disease resistance effects (10–12), has attracted much attention in recent years. Eugenol is a phenolic aromatic compound, which is the main component of clove oil (13). It is commonly obtained from the natural essential oils of the *Lamiaceae*, *Lauraceae*, *Myrtaceae* and *Myristicaceae* families (14). Recent reports indicate that eugenol inhibits LPS-induced inflammatory response in the porcine intestinal epithelial cells (15). In addition, studies have shown that eugenol has antiviral activity against Ebola virus (a single-stranded, negative-sense, enveloped filamentous RNA virus) (16) and feline calicivirus (a single-stranded, positive-sense, non-enveloped RNA virus) (17), but the specific mechanism awaits study. Besides, little is known about its physiological function in porcine intestinal epithelial cells. More importantly, no previous study has investigated the key regulatory functions of eugenol during TGEV infection.

Thus, in the present study, we focused on the effects of eugenol on intestinal epithelial function and inflammatory response in TGEV-infected weaned piglets and revealed the underlying mechanism. Our findings suggested that eugenol protects intestinal epithelial barrier function by inhibiting TGEV-induced intestinal epithelial cell inflammation, and the mechanism is related to the inhibition of TGEV-induced *NF-κB* signaling pathway.

## Materials and methods

### Materials

Eugenol (≥98%, W246719, FG) was acquired from Sigma-Aldrich (Shanghai, China). NF-κB inhibitor (BAY 11-7082, S2913) was purchased from Selleck.

### Virus, cell culture, and treatment

TGEV strain TS (GenBank accession no. DQ201447.1), a clinical isolate, was presented by the College of Veterinary Medicine, Sichuan Agricultural University. IPEC-J2 (Porcine Small Intestinal Epithelial Cell Line) cells were purchased from the American type culture collection (ATCC, ACC 701). IPEC-J2 cells were cultured in DMEM-F12 (Gibco, Shanghai, China) including 10% fetal bovine serum (Gibco, Shanghai, China) and 1% streptomycin and penicillin (Gibco, Shanghai, China) in a humidified incubator at 37 °C, 5% CO_2_. Eugenol (200 μM) was incubated with cells for 1 h before exposure to TGEV (MOI=1); thereafter IPEC-J2 cells were incubated with eugenol. The cells were pretreated with BAY-117082 (1 μM) for 1 h before adding eugenol and TGEV.

### Experimental design and diet

**Figure 1:**
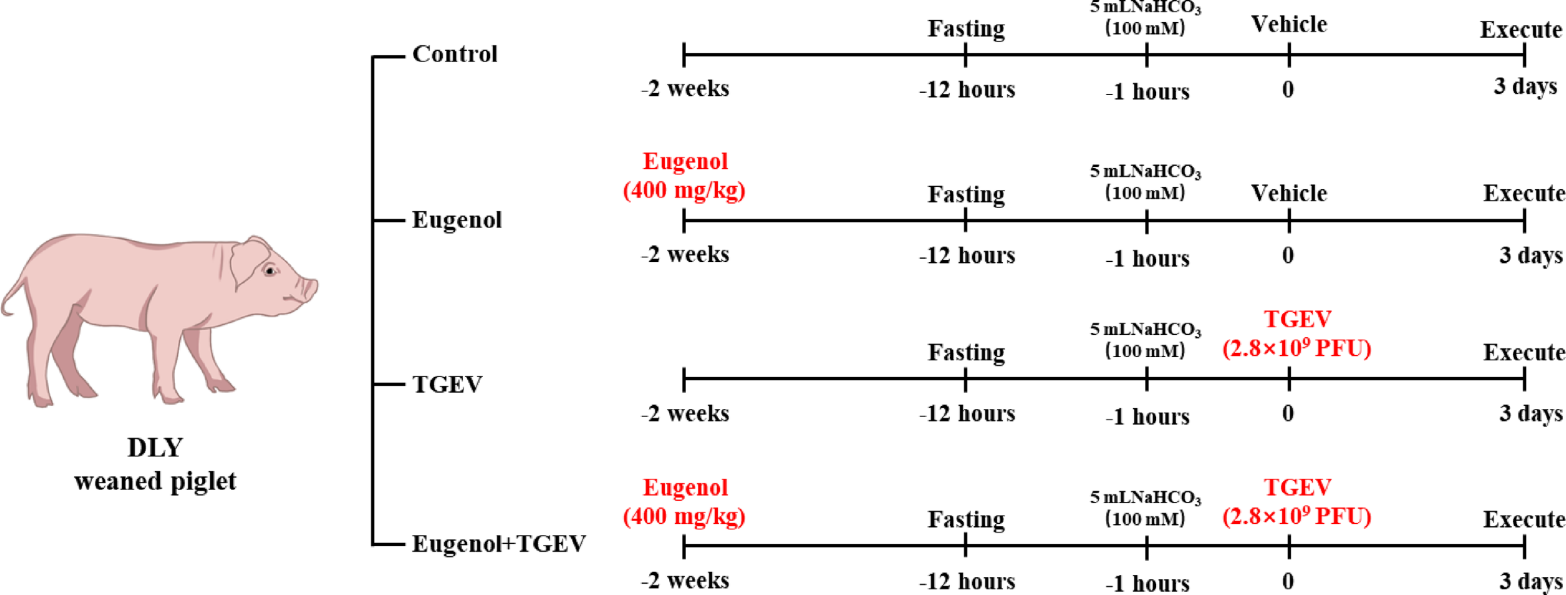
Design diagram of experimental piglets.

All animal experiments were approved by the Institutional Animal Care and Use Committee of the Laboratory Animal Center at Sichuan Agricultural University (SICAU-2015-033). Twenty-one-day-old DLY weaned piglets were obtained from a pig farm in Mianyang, Sichuan, China. Thirty-two piglets were randomly divided into four groups of equal weight: (1) control group (piglets fed with basal diet); (2) eugenol supplemented group (piglets fed with basal diet containing 400 mg/kg eugenol); (3) TGEV-infected group (piglets fed with basal diet); (4) eugenol +TGEV-infected group (piglets fed with basal diet containing 400 mg/kg eugenol). There were 8 replicate piglets per treatment. The basal diet was formulated to meet the swine nutrient requirements recommended by National Research Council (NRC, 2012). Piglets were fed a basal diet for 3 days before the trial began. As shown in **Figure 1**, on day 15 of the formal trial, after 11 hours of fasting, each pig received 5 ml 100 mM NaHCO_3_ to neutralize gastric acid and avoid acidic environment to affect virus viability. One hour later, 0 or 2.8×10^9^ PFU TGEV (TCID50 = 10 ^-6.67^/100 μL) was administered according to the group. All piglets were executed on day 18 to collect samples.

### Diarrhea rate

Daily diarrhea rate records were performed on piglets according to **Table 1**. Piglets with a score of 3, 4 or 5 were considered to have diarrhea. The diarrhea rate of each piglet was calculated as follows:

**Table 1:** Fecal scores standard.

Diarrhea degree	Excrement shape	Excrement score
Normal	Hard bar/Hard granulous	1
Normal	Firm well formed	2
Light	Soft/Forming	3
Middle	Dense/Not formed	4
Severity	Fluid/Not formed	5

Diarrhea rate (%) = (days of piglet diarrhea/total days) × 100.

### Sample collection

On day 18 of the formal trial, blood samples were collected from the anterior vena cava of each piglet. The whole blood was placed in vacuum collection vessel and stood at room temperature for 30min. The serum samples were centrifuged at 3000 r/min for 10 min at 4°C and then separated into 200 μL eppendorf tubes. The serum samples were stored at -20°C. The piglets were euthanized after blood collection and slaughtered promptly. After opening the abdominal cavity of the piglets, the middle duodenum, jejunum and ileum tissues were selected for fixation with 4% paraformaldehyde solution, and then morphological analysis was performed. The middle jejunum tissue was selected for longitudinal incision, rinsed with normal saline, scraped the intestinal mucosa, placed in a sterile cryopreservation tube, and stored at -80°C until analysis.

### Serum inflammatory cytokines and immunoglobulin detection

The concentration of inflammatory cytokines (*IL-1β* and *TNF-α*) and immunoglobulin (*IgA* and *IgG*) in serum were determined, following the instructions of a commercially available porcine Enzyme-Linked Immunosorbent Assay (ELISA) kits (Shanghai Meimian Biotechnology, Shanghai, China). All procedures were guided by manuals of the kits. For quantification, the standards provided in the kits were used to generate standard curves.

### Histomorphology analysis of intestinal segments

Intestinal samples were collected and fixed in 4% paraformaldehyde. Tissues were dehydrated by a series of alcohols transferred at increased concentrations. Then the duodenum, jejunum and ileum sections were embedded in paraffin and cut into 5μm thick sections for hematoxylin and eosin staining. At least 10 villi with intact lamina propria from each sample were blindly selected and examined for measurement. Image Pro Plus 6.0 was used to measure the height of 10 intact villi and the corresponding crypts in intestinal tissue, and calculate the villus-crypt ratio. Two observers viewed and evaluated slices.

### Western blotting and RT-PCR

The protein content of the intestinal tissues and cells was measured using the BCA Protein Assay Kit (Thermo Fisher, 23225) after they were lysed in RIPA buffer. Equivalent amounts of each protein extract were separated on 8%, 10% or 12% SDS-polyacrylamide gel electrophoresis (SDS-PAGE). After electrophoresis, the gel with separated proteins was released from the glass plate and those protein samples were electrophoretically transferred onto polyvinylidene difluoride (PVDF) membrane (Millipore, Billerica, MA, USA). After transfer, PVDF membranes were placed on a shaker and block with 5% skim milk powder at room temperature for 90 min. After blocking, membranes were treated with the appropriate primary antibodies overnight at 4 °C, including *ZO-1* (proteintech, 1773-1-AP), *Claudin-1* (proteintech, 13050-1-AP), *Occludin* (proteintech, 27260-1-AP), *NF-κB p65* (CST, 8242), *Phospho-NF-κB p65* (CST, 2928), *β-actin* (CST, 3700) antibodies. The dilution ratio of primary antibodies was 1:1000. After 3 times rinsing with Tris-Buffered-Saline with Tween (TBST), the membrane was incubated with horseradish peroxidase (HRP)-conjugated secondary antibodies for 1 h at room temperature. Immuno-reactive proteins were visualized using a chemiluminescence detection kit (Beyotime, P0018FS). An imaging system (ChemiDoc) and Image Lab software were used to determine the blot signal and protein density.

According to the manufacturer’s manual, total RNA was extracted from tissues and cells samples using Trizol reagent (Takara Bio, 9109). Both genomic DNA removal and reverse transcription were performed using PrimeScript RT reagent kit with gDNA eraser (Takara Bio, RR047A), following the manufacturer’s guidelines. All primers are validated by Blast analysis prior to RT-PCR (**Table S1**). The PCR procedure was as follows: pre-denaturating at 95 °C for 1 min, 40 cycles of denaturation at 95 °C for 15 s, annealing at 60 °C for 30 s, and extension at 95 °C for 15 s and a cycle of final extension at 72 °C for 6 min. The generated Gene-specific amplification products were confirmed by melting curve analysis after each real-time quantitative PCR assay. The specificity of the reaction was confirmed by verifying the expected size of the PCR product on 2% agarose gel. The relative gene expression was calculated by 2 ^-ΔΔCT^ method. *β-actin* was used as the housekeeping gene.

### Statistical analysis

GraphPad Prism 8.0 software was used for data analysis. Data are presented as mean ± standard error of mean (SEM). The chi-square test was used to test for diarrhea rate. The t-tests (two-tailed) was used for growth performance of piglets before TGEV infection, and one-way ANOVA with Dunnett’s multiple comparisons test was used for other results. *P* values < 0.05 were considered statistically significant.

## Results

### Effects of eugenol on growth performance and diarrhea rate in TGEV-infected weaned piglets

We first evaluated the effect of eugenol on growth performance and diarrhea rate in TGEV-infected piglets. As shown in **Table 2**, compared with the control group, TGEV infection significantly reduced F/G (*P* < 0.01), but the eugenol supplementation significantly improved the F/G of weaned piglets (*P* < 0.01). In TGEV-infected piglets, eugenol significantly increased piglet ADG (*P* < 0.01). In addition, TGEV challenge induced severe diarrhea in piglets, and the diarrhea rate was significantly increased (*P* < 0.01), while eugenol supplementation reduced the diarrhea rate in piglets (*P* < 0.05). To determine the success of TGEV infection, we also assessed the expression of TGEV-N mRNA in jejunum mucosa (**Figure S1**). The results showed that eugenol significantly reduced the replication of TGEV in jejunum of weaned piglets after TGEV was successfully infected (*P* < 0.01).

**Table 2:** The effects of eugenol and/or TGEV challenge on growth performance and diarrhea of weaned piglets.

Items	Treatment				*P-*value			
	CON	EUG	TGEV	T+EUG	SEM	EUG	TGEV	EUG*TGEV
1-14 d								
ADFI (g)	244.3	250.6			15.73	0.69		
ADG (g)	127.4	157.1*			14.54	<0.05		
F/G	1.87	1.51*			0.11	<0.01		
Diarrhea rate (%)	22.32	9.375*			5.89	<0.05		
15-18 d								
ADFI (g)	388.4	407.1	407.4	403.3	12.38	0.76	0.77	0.66
ADG (g)	214.8^a^	265.8^a^	151.9^b^	229.2^a^	11.21	<0.01	<0.01	0.47
F/G	1.86^b^	1.56^b^	2.73^a^	1.85^b^	0.1	<0.01	<0.01	0.04
Diarrhea rate (%)	11.11^b^	0^b^	51.85^a^	25^b^	4.82	0.01	<0.01	0.28

ADFI average daily feed intake, ADG average daily gain, F/G Feed/Gain ratio.

^1^* means significant difference in peer data between CON and EUG groups, P < 0.05.

^2^a, b mean values within a row with unlike superscript letters were significantly different, P < 0.05.

^3^CON, piglets were fed with basal diet; EUG, piglets were fed with basal diet containing 400 mg/kg eugenol; TGEV, piglets fed with basal diet and infected by TGEV; T+EUG, piglets were fed with basal diet containing 400 mg/kg eugenol and infected by TGEV.

### Effects of eugenol on serum inflammatory factors and immunoglobulin contents in TGEV-infected weaned piglets

**Figure 2:**
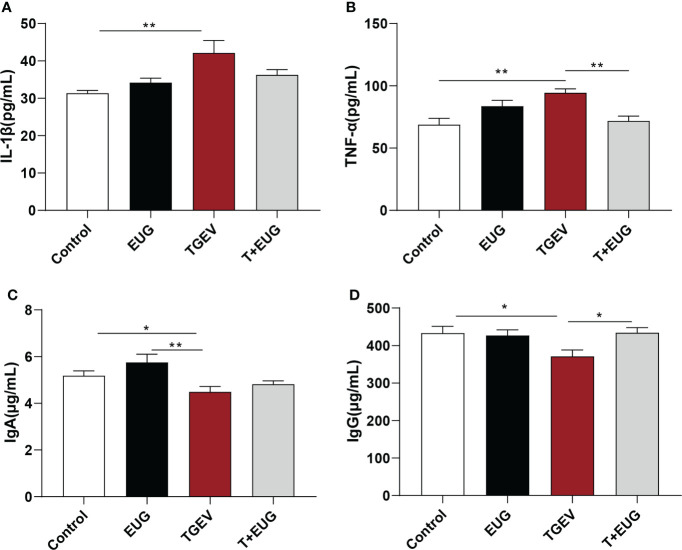
Effects of eugenol on serum inflammatory factors and immunoglobulin contents in TGEV-infected weaned piglets. **(A–D)** The levels of *IL-1β*, *TNF-α*, *IgA* and *IgG* in serum were detected by ELISA. Data were expressed as mean ± SEM. ^*^ *p* < 0.05, ^**^ *p* < 0.01.

The severity of viral infection depends on the development of a cytokine storm characterized by elevated serum levels of inflammatory cytokines (18). As shown in **Figure 2**, compared with the control group, TGEV challenge significantly increased the levels of *IL-1β* and *TNF-α* in the serum of piglets (*P* < 0.01), and significantly decreased the levels of *IgA* and *IgG* in the serum (*P* < 0.05). Under the condition of TGEV challenge, eugenol supplementation significantly reduced the level of *TNF-α* in serum (*P* < 0.01) and increased the content of *IgG* in serum (*P* < 0.05).

### Effects of eugenol on jejunum inflammation-related indexes in TGEV-infected weaned piglets

**Figure 3:**
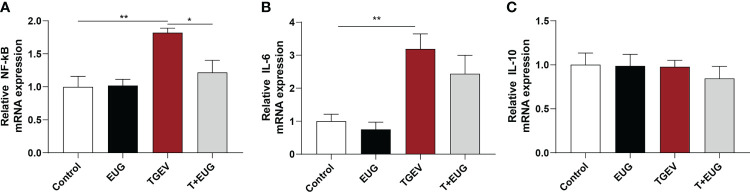
Effects of eugenol on jejunum inflammatory-related genes expression levels in TGEV-infected weaned piglets. **(A-C)** Levels of *NF-κB*, *IL-6* and *IL-10* mRNA in the jejunum mucosa was examined by RT-PCR. Data were expressed as mean ± SEM. ^*^ *p* < 0.05, ^**^ *p* < 0.01.

**Figure 4:**
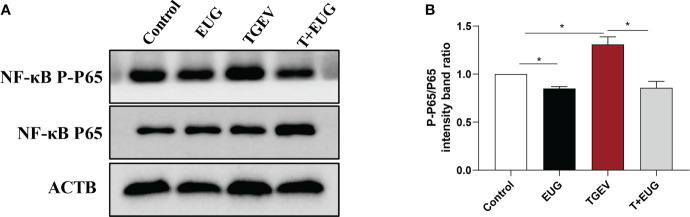
Effects of eugenol on jejunum inflammatory-related protein expression levels in TGEV-infected weaned piglets. **(A, B)** Immunoblot analysis and quantification of *NF-κB P-P65* and *NF-κB P65* in jejunum mucosa. Data were expressed as mean ± SEM. ^*^ *p* < 0.05.

Increased and sustained *NF-κB* activation induces inflammation and tissue damage (19). As shown in **Figure 3**, compared with the control group, TGEV infection significantly increased the relative expression of *NF-κB* and *IL-6* mRNA in the jejunum of weaned piglets (*P* < 0.01). Under the condition of TGEV challenge, eugenol supplementation significantly alleviated the TGEV-induced increase in the relative expression of *NF-κB* mRNA (*P* < 0.05). In addition, as shown in **Figure 4**, compared with the control group, TGEV infection significantly increased the protein expression level of *NF-κB P-P65* in the jejunum of weaned piglets (*P <*0.05), and dietary supplementation of eugenol reduced the protein expression level of *NF-κB P-P65* in the jejunum of piglets (*P <*0.05). Interestingly, eugenol supplementation could significantly alleviate the TGEV-induced increase in the protein expression level of *NF-κB P-P65* (*P <*0.05).

### Effects of eugenol on small intestine morphology in TGEV-infected weaned piglets

**Figure 5:**
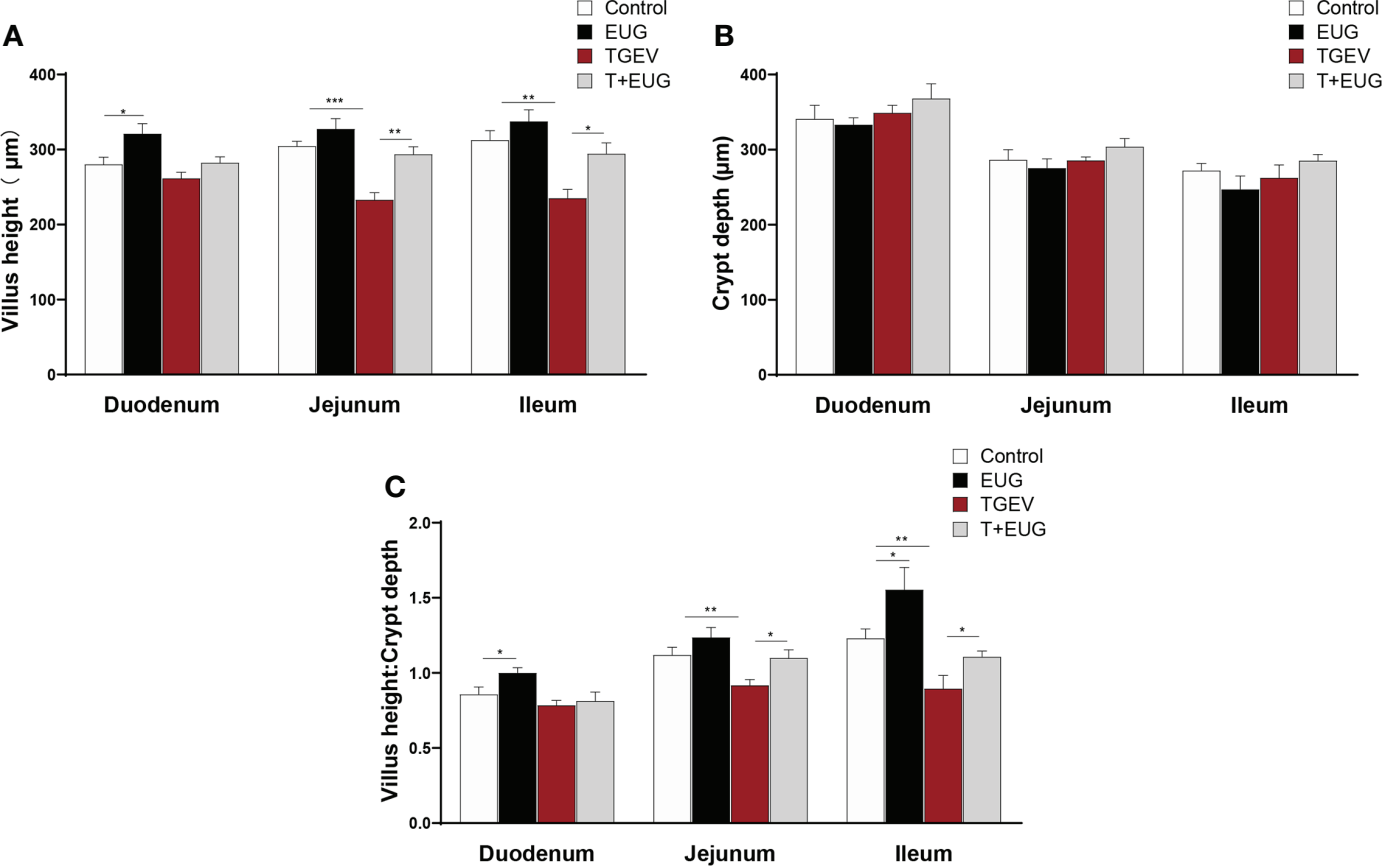
Effects of eugenol on small intestine morphology in TGEV-infected weaned piglets. Data were expressed as mean ± SEM. ^*^ *p* < 0.05, ^**^ *p* < 0.01, ^***^ *p* < 0.001.**(A–C)** Statistical analysis of villus height, crypt depth and villus height to crypt depth ratio in small intestine.

Important indicators for evaluating the absorptive function of the small intestine are villus height, crypt depth, and V/C (the ratio of villus height to crypt depth) (20). As shown in **Figure 5**, compared with the control group, eugenol supplementation significantly increased the duodenum villus height and the ratio of villous height to crypt depth of weaned piglets, as well as the ileum the ratio of villous height to crypt depth (*P* < 0.05); TGEV infection significantly decreased the villus height and the ratio of villous height to crypt depth in the jejunum and ileum of weaned piglets (*P* < 0.01). Moreover, eugenol supplementation alleviated TGEV-induced reduction of the ratio of villous height to crypt depth in jejunum and ileum (*P* < 0.05).

### Effects of eugenol on serum D-lactic acid concentrations in TGEV-infected weaned piglets

**Figure 6:**
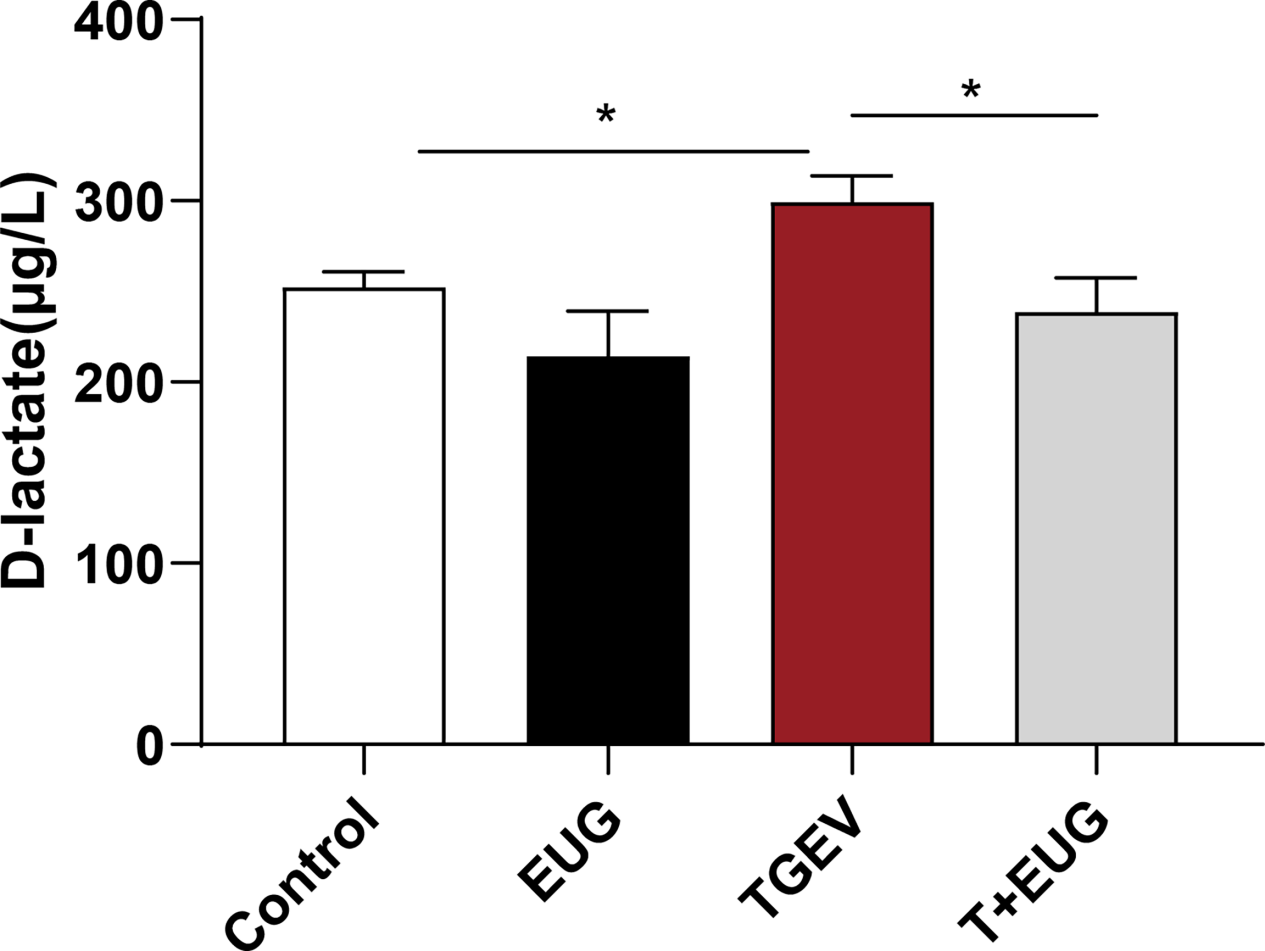
Effects of eugenol on serum D-lactic acid concentrations in TGEV-infected weaned piglets. The levels of D-lactic acid in serum were detected by ELISA. Data were expressed as mean ± SEM. ^*^ *p* < 0.05,

After the intestinal injury, D-lactic acid in the intestine will enter the peripheral blood through the intestinal mucosa (21). As shown in **Figure 6**, compared with the control group, TGEV infection significantly increased the D-lactic acid content in the serum of weaned piglets (*P <*0.05). In addition, eugenol supplementation significantly inhibited the increase of D-lactic acid content induced by TGEV (*P <*0.05).

### Effects of eugenol on jejunum intestinal barrier related proteins in TGEV-infected weaned piglets

**Figure 7:**
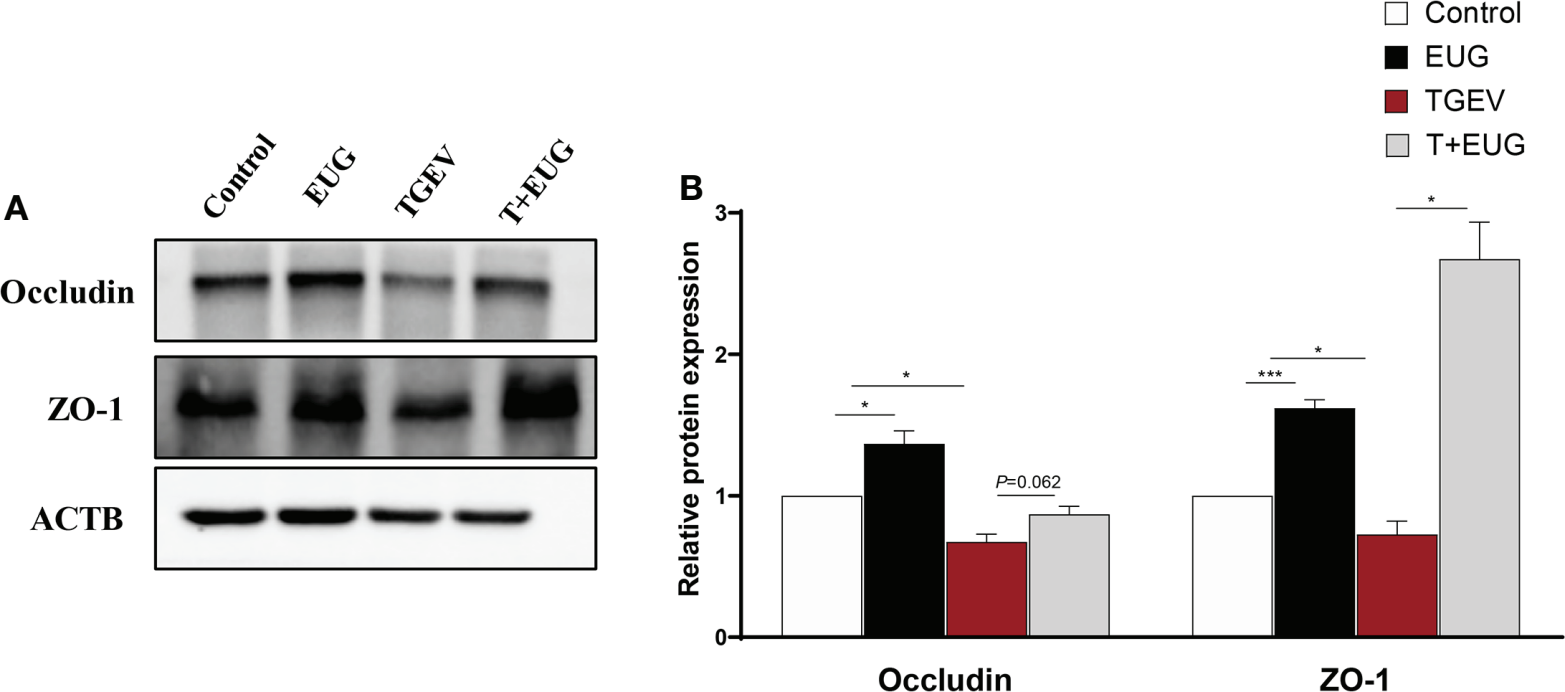
Effects of eugenol on jejunum intestinal barrier related proteins expression levels in TGEV-infected weaned piglets. **(A, B)** Immunoblot analysis and quantification of *ZO-1* and *Occludin* in jejunum mucosa. Data were expressed as mean ± SEM. ^*^ *p* < 0.05.^***^ *p* < 0.001

Tight junctions (TJs) are one kind of cell−cell adhesion complex that connects cells and provides seal around cells (22). As shown in **Figure 7**, compared with the control group, TGEV infection significantly reduces the protein expression levels of *ZO-1* and *Occludin* in the jejunum of weaned piglets (*P* < 0.05), and eugenol supplementation increase the protein expression levels of *ZO-1* (*P* < 0.001) and *Occludin* (*P* < 0.05). In addition, eugenol supplementation significantly alleviated the reduction of *ZO-1* protein expression induced by TGEV infection (*P* < 0.05), and had a tendency to alleviate the protein expression of *Occludin* (*P* = 0.062).

### Effects of eugenol on jejunum transporte related genes expression levels in TGEV-infected weaned piglets

**Figure 8:**
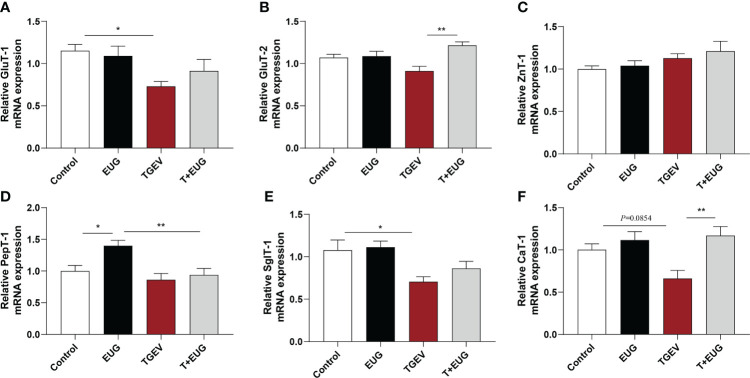
Effects of eugenol on jejunum transporte related genes expression levels in TGEV-infected weaned piglets. **(A-F)** Levels of *GluT-1*, *GluT-2*, *ZnT-1*, *PepT-1*, *SglT-1* and *CaT-1* mRNA in the jejunum mucosa was examined by RT-PCR. Data were expressed as mean ± SEM. ^*^ *p* < 0.05, ^**^ *p* < 0.01.

As shown in **Figure 8**, compared with the control group, TGEV infection significantly reduces the relative expression of *GluT-1* and *SglT-1* mRNA in the jejunum of weaned piglets (*P*<0.05), and the relative expression of *CaT-1* mRNA has a tendency to decrease (*P*=0.0854). In addition, eugenol supplementation significantly increased the relative expression of *PepT-1* mRNA in the jejunum of piglets (*P*<0.05). Under the condition of TGEV infection, eugenol supplementation significantly increases the relative expression of *GluT-2* and *CaT-1* mRNA (*P*<0.01).

### Effects of TGEV infection on intestinal barrier injury of IPEC-J2 cells

**Figure 9:**
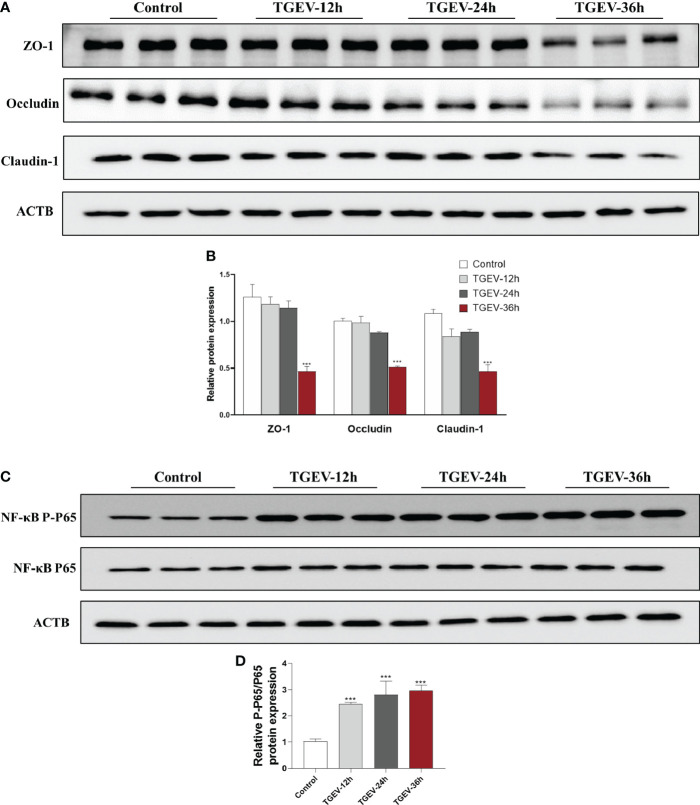
Effects of TGEV infection on intestinal barrier injury of IPEC-J2 cells. IPEC-J2 cells were infected with TGEV (MOI=1) for 12 h, 24 h and 36h. **(A-D)** Immunoblot analysis and quantification of *ZO-1*, *Occludin*, *Claudin-1, NF-κB P-P65 and NF-κB P65* in IPEC-J2 cells. Data were expressed as mean ± SEM. ^***^ *p* < 0.001.

To further elucidate the underlying mechanism of eugenol to alleviate TGEV infection, we used TGEV to infect IPEC-J2 cells to construct an *in vitro* infection model. The effects of TGEV (MOI=1) infection on IPEC-J2 cells for 12, 24 and 36 h on the tight junction and inflammation proteins are shown in **Figure 9**. Compared with the control group, the protein expressions of *ZO-1*, *Occludin* and *Claudin-1* were significantly decreased by TGEV infection for 36h in IPEC-J2 cells (*P* < 0.001), and there was a certain time effect. In addition, TGEV infection promoted the phosphorylation of *NF-κB P65* protein at 12, 24 and 36h (*P* < 0.001), thus inducing the IPEC-J2 cells inflammatory response.

### Effects of TGEV infection on the morphology of IPEC-J2 cells

**Figure 10:**
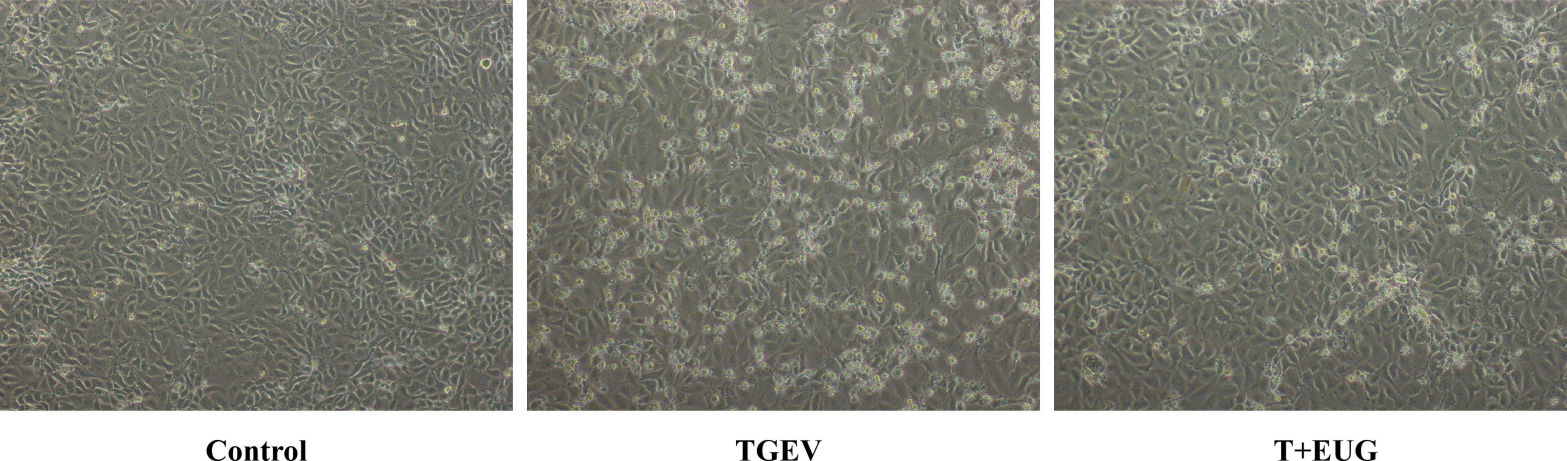
Effects of TGEV infection on the morphology of IPEC-J2 cells. Phase-contrast images of TGEV-treated IPEC-J2 cells. Scale bar, 50 μm.

*In vitro*, we observed the effect of TGEV infection on IPEC-J2 cell morphology. As shown in **Figure 10**, compared with the control group, TGEV infection had a cytopathic effect on IPEC-J2 cells. Eugenol treatment was able to reverse TGEV-induced cytopathic effects.

### Eugenol alleviates TGEV-induced intestinal barrier damage and inflammation in IPEC-J2 cells

**Figure 11:**
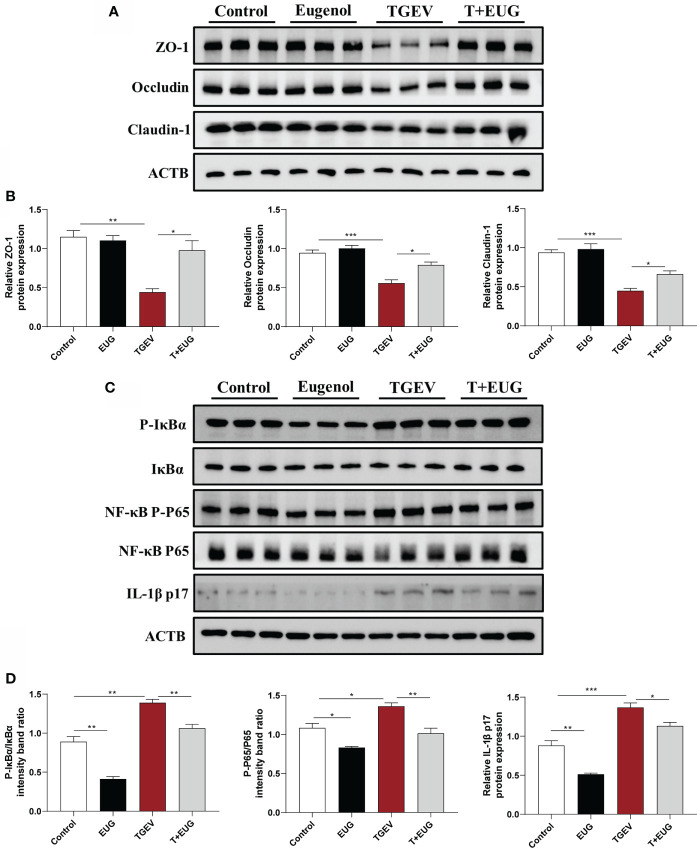
Eugenol alleviates TGEV-induced intestinal barrier damage in IPEC-J2 cells. IPEC-J2 cells were pretreated with eugenol (200 μM) for 1 h and then infected with TGEV for 36 h. **(A, B)** Immunoblot analysis and quantification of *ZO-1*, *Occludin* and *Claudin-1* in IPEC-J2 cells. **(C, D)** Immunoblot analysis and quantification of *P-IκBα*, *IκBα*, *NF-κB P-P65*, *NF-κB P65* and *IL-1β* in IPEC-J2 cells. Data were expressed as mean ± SEM. ^*^ *p* < 0.05, ^**^ *p* < 0.01, ^***^ *p* < 0.001.

Consistent with *in vivo* experiments, as shown in **Figure 11**, the addition of eugenol significantly alleviated the TGEV-induced decrease in the protein expressions of *ZO-1*, *Occludin* and *Claudin-1* in IPEC-J2 cells (*P* < 0.05). Based on these data, we further validated the anti-inflammatory ability of eugenol *in vitro*. The addition of eugenol significantly alleviated the TGEV-induced increase in the relative protein expression of *P-IκBα*/*IκBα*, *NF-κB P-P65*/*P65* and *IL-1β P17* in IPEC-J2 cells (*P* < 0.05).

### Eugenol alleviates TGEV-induced intestinal barrier dysfunction through the *NF-κB* signaling pathway.

**Figure 12:**
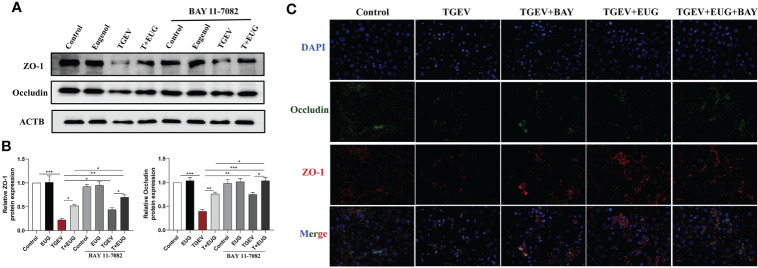
Eugenol alleviates TGEV-induced intestinal barrier dysfunction through the NF-κB signaling pathway. IPEC-J2 cells were pretreated with eugenol (200 μM) or BAY 11-7082(1 μM) for 1 h and then infected with TGEV for 36 h. **(A, B)** Immunoblot analysis and quantification of *ZO-1* and *Occludin* in IPEC-J2 cells. **(C)** Representative images of *ZO-1* and *Occludin* by immunofluorescence staining in IPEC-J2 cells. Scale bar, 50 μm. Data were expressed as mean ± SEM. ^*^ *p* < 0.05, ^**^ *p* < 0.01, ^***^ *p* < 0.001.

To further explore the mechanism of eugenol attenuating TGEV-induced intestinal barrier damage, we used an *NF-κB* inhibitor (BAY 11-7082) to analyze whether TGEV-induced intestinal barrier damage could be alleviated by inhibiting inflammation. As shown in **Figure 12**, BAY11-7082 treatment significantly inhibited the TGEV-induced reduction of *ZO-1* and *Occludin* protein level (*P* < 0.05). In addition, eugenol-promoted intestinal barrier restoration was significantly enhanced by BAY 11-7082, as evidenced by increased proteins in *ZO-1* and *Occludin*; this result was confirmed by immunofluorescent staining refer to **Figure 12C**.

## Discussion

During weaning, piglets experience pathogen proliferation, diarrhea, poor growth performance and even death due to the transition from breast milk to solid feed (23, 24). TGE was a highly contagious infectious disease caused by TGEV causing viral diarrhea in weaned piglets. Numerous studies have pointed out that plant extracts play an important role in promoting animal growth performance, enhancing immunity, and maintaining animal health (25–27). At present, there are few studies on the growth performance and immune regulation of eugenol in piglets, and a large number of studies focus on the effect of mixed essential oils (28, 29). Therefore, we used a single ingredient, eugenol, for the trial study. Studies have shown that compared with the control group, the ADG of piglets supplemented with eugenol increases by 17.18-30.08% (30). Here, we showed that eugenol supplementation significantly improved ADG and F/G of weaned piglets, and reduced diarrhea rate of piglets. These results indicated that eugenol supplementation relieves weaning stress of piglets at weaning stage. Eugenol has important reference significance as an antibiotic substitute for piglets during the period of antibiotic prohibition. In addition, previous research found that TGEV infection significantly reduces growth performance and causes severe diarrhea in weaned piglets (31, 32), which is consistent with our results. We found that under TGEV infection, the addition of eugenol significantly alleviates the TGEV-induced decline in growth performance and reduce the diarrhea rate of piglets. Therefore, our results suggested that eugenol alleviates the infection symptoms of TGEV in weaned piglets.

TGEV infection induces the expression of inflammatory factors, aggravates intestinal damage, and causes damage to the intestinal barrier (33). We found that TGEV infection significantly increased the levels of *IL-1β* and *TNF-α* in the serum of piglets, increased the mRNA expressions of *NF-κB* and *IL-6* in the jejunum of piglets, and aggravated the phosphorylation level of *NF-κB P-P65* protein. These data suggested TGEV infection may induce the overexpression of inflammatory markers in weaned piglets, thereby causing the immune response disorder in weaned piglets. Furthermore, under the condition of TGEV infection, eugenol alleviated the *TNF-α* concentration in serum, the *NF-κB* mRNA expression and the hyper-phosphorylation of *NF-κB P65* protein in jejunum. Consistent with previous studies *in vivo* (34), eugenol possesses a strong anti-inflammatory ability, suggesting that eugenol alleviated intestinal excessive inflammatory response caused by TGEV infection. However, we found that *IL-1β* was not significantly reduced by eugenol. The failure of eugenol to significantly reduce *IL-1β* may be related to the potential infection sites of the virus and the action sites of eugenol. Whether TGEV induces inflammation in organs and systems such as the liver, lungs and nervous system, and whether eugenol plays an anti-inflammatory role in these organs and systems are unknown. These areas require further investigation. *IgA* and *IgG* are important components of adaptive immunity and are involved in a variety of immune functions, including protection from microbial infection, humoral immunity, and immune homeostasis (35–37). IgA, the most abundantly produced antibody isotype in mammals (38), maintains the homeostasis of the mucosal surfaces of the gastrointestinal tract, and protects these surfaces from viral infection (39). Our study showed that TGEV infection significantly reduced the levels of *IgA* and *IgG* in the serum of weaned piglets, which indicated that TGEV infection not only induced excessive innate immunity in piglets, but also disturbed the adaptive immunity of piglets. Supplementation of plant extract cinnamaldehyde enhanced the acquisition of specific antibodies during helminth infection (40). In this study, eugenol increases the level of *IgG* in serum of TGEV-infected piglets, indicating that plant essential oils improve the body’s immune function during pathogen infection.

Small intestine is mainly to digest ingested food and absorb nutrients (41). Villus height, crypt depth, and the ratio of villus height to crypt depth are important indicators for evaluating the absorptive function of the small intestine (42). Under normal physiological conditions, intestinal villous epithelial cells slough off normally. The exfoliated cells migrate from the base of the crypts, which in turn differentiate and give rise to mature villi cells to the ends of the villi (43). Here, we found that TGEV infection reduced the villus height and the ratio of villus height to crypt depth in the jejunum and ileum of weaned piglets, and eugenol supplementation alleviate the damage of TGEV on the intestinal structure of piglets. D-lactic acid is a bacterial metabolite produced by gut flora (44). When intestinal permeability is abnormally increased due to some disruption, D-lactic acid in the intestinal lumen easily enters the peripheral blood through the intestinal mucosa (45). In the present study, the serum D-lactic acid level was increased in TGEV-infected piglets, and eugenol significantly inhibits the TGEV-induced increase in D-lactic acid content.

Tight junctions, the main connection mode between intestinal mucosal epithelial cells, maintain the integrity of the intestinal mucosal barrier mechanical structure and function (46). A large number of *in vitro* experiments proved that TGEV reduces the protein levels of *Claudin-1*, *Occludin* and *ZO-1* in IPEC-J2 cells (47, 48), which is closely related to its cause of viral enteritis, diarrhea and morbidity in piglets. This experimental study showed that TGEV infection of weaned piglets reduces the protein expression levels of *ZO-1* and *Occludin*; while eugenol alleviates the reduction of *ZO-1* protein expression levels induced by TGEV infection. This indicated that eugenol alleviates the intestinal barrier function damage caused by TGEV infection by promoting the expression of intestinal tight junction protein.

Glucose is one of the most important energy sources in animals, and *GluT1* is a uniporter protein that is located on the cell membrane or cell surface and helps transport glucose into mammalian cells (49). The sustained expression of *GluT1* protein enables efficient glucose transport and glucose utilization, and then glucose is absorbed by the active sugar transporter *SglT1* at the brush border of intestinal epithelial cells (50). In recent years, oligopeptide transporter 1 (*PepT1*) was found to play a key role in intestinal homeostasis in metabolite profiling and tissue physiology (51). *PepT1* is predominantly expressed in the small intestine and transports dipeptides/tripeptides for metabolic purposes (52). The family of cationic amino acid transporters (CaT, slc7a), called system y^+^, transports cationic amino acids such as L-lysine, L-histidine, L-ornithine and L-arginine. *CaT1* is considered a key component of the y^+^ transport system, with transport characteristics including independence for sodium and pH and preference for cationic amino acids as substrates (53, 54). We found that dietary supplementation of eugenol could significantly increase the relative expression of *PepT1* mRNA and promote the metabolism of dipeptide/tripeptide in piglets, which may be related to the increase in feed efficiency in piglets. TGEV infection reduces the relative expression of *GuT-1* and *SglT-1* mRNA in the jejunum of weaned piglets, and the relative expression of *CaT-1* mRNA has a tendency to decrease, indicating that TGEV infection seriously reduces the intestinal glucose transport and absorption function, and cationic amino acid transport function of weaned piglets. However, eugenol supplementation reversed the TGEV-induced decrease of *GLUT-2* and *CAT-1* mRNA relative expression levels. Overall, the interaction of the intestinal chemical barrier, immune barrier and physical barrier jointly maintains the homeostasis of the body of weaned piglets and ensures that the small intestine can fully perform its functions of digestion and absorption.

Intestinal barrier dysfunction and immune disorders are two essential factors affecting the pathogenesis of intestinal diseases (55, 56). In general, intestinal epithelial tight junction act as a physical barrier limiting mucosal immune system exposure to pathogenic microorganisms (8). However, accumulating evidence confirms that inflammation is an important trigger for intestinal barrier disruption (57, 58). Our results suggested that TGEV infection reduces the protein expression of *ZO-1*, *Occludin* and *Claudin-1* and increased the phosphorylated protein expression level of *NF-κB p65* in IPEC-J2 cells, which was similar to recent studies (33). This suggested that TGEV may induce an excessive inflammatory response in IPEC-J2 cells to induce intestinal barrier damage. Meanwhile, consistent with the *in vivo* results, eugenol treatment increases the protein levels of *ZO-1*, *Occludin* and *Claudin*-1, and decreases the phosphorylated protein levels of *IκBα* and *NF-κB* and the activation of *Il-1β* in TGEV-infected IPEC-J2 cells. Il-1β was reported to reduce the expression of *ZO-1* and *Occludin* in CACO-2 cells (59). Thus, the activity of relevant inflammatory factors may influence intestinal barrier function. To further explore the crosstalk between inflammation and the intestinal barrier, we treated IPEC-J2 cells with BAY 11-7082, a specific inhibitor of *NF-κB*. We found that BAY 11-7082 attenuates TGEV-induced intestinal barrier damage. Emerging evidence suggested that inhibition of *NF-κB* expression in intestinal epithelial cells alleviates TNF-α/interferon-γ-induced intestinal barrier disruption (60). Therefore, our results suggested that eugenol may inhibit TGEV-induced intestinal injury through the *NF-κB* pathway. However, little is known about inhibition of NF-κB signaling or eugenol regulation of tight junction expression, since effector molecules and receptors remain unknown. Therefore, more studies on how eugenol regulates tight junction protein expression are needed in the future.

In conclusion, eugenol supplementation can alleviate the damage to the intestinal structure and the disturbance of intestinal immune functions of piglets caused by TGEV, and improve intestinal transit, digestion and absorption function in piglets. Eugenol’s role in improving intestinal barrier function may provide a potential approach to repair intestinal barrier dysfunction caused by viral infection.

## Data availability statement

The datasets presented in this study can be found in online repositories. The names of the repository/repositories and accession number(s) can be found in the article/**Supplementary Material**.

## Ethics statement

The animal study was reviewed and approved by Institutional Animal Care and Use Committee of the Laboratory Animal Center at Sichuan Agricultural University(SICAU-2015-033).

## Author contributions

KW and JL conceived and designed the experiments. KW performed cell experiments and animal experiments and wrote the manuscript. DC, BY, JH, XM, ZH, HY, YL, JY and PZ performed biochemical analysis. AW, JL, XM, and HY gave constructive comments for the results and discussion of the manuscript. All authors have read and approved the final manuscript.

## Funding

This work was supported by National Natural Science Foundation of China (NSFC) (31702124), and Natural Science Foundation of Sichuan Province (2022NSFSC0072).

## Acknowledgments

We thank Xiu Wu, Hongmin Liang and Yang Tang for their assistance during the experiments.

## Conflict of interest

The authors declare that the research was conducted in the absence of any commercial or financial relationships that could be construed as a potential conflict of interest.

## Publisher’s note

All claims expressed in this article are solely those of the authors and do not necessarily represent those of their affiliated organizations, or those of the publisher, the editors and the reviewers. Any product that may be evaluated in this article, or claim that may be made by its manufacturer, is not guaranteed or endorsed by the publisher.

## References

[1] Reyna VF. Of viruses, vaccines, and variability: Qualitative meaning matters. Trends Cognit Sci (2020) 24:672–5. doi: 10.1016/j.tics.2020.05.015 PMC7266748 32600966

[2] Turlewicz-Podbielska H Pomorska-Mól M. Porcine coronaviruses: Overview of the state of the art. Virol Sin (2021) 36:833–51. doi: 10.1007/s12250-021-00364-0 PMC7959302 33723809

[3] Wu A Yu B Zhang K Xu Z Wu D He J. Transmissible gastroenteritis virus targets paneth cells to inhibit the self-renewal and differentiation of Lgr5 intestinal stem cells *via* notch signaling. Cell Death Dis (2020) 11:40. doi: 10.1038/s41419-020-2233-6 31959773 PMC6971083

[4] Zhang L Qin Y Chen M. Viral strategies for triggering and manipulating mitophagy. Autophagy (2018) 14:1665–73. doi: 10.1080/15548627.2018.1466014 PMC6135629 29895192

[5] Leopoldt D Meyer U. Die transmissible gastroenteritis der schweine als modell für infektionsbedingte durchfallerkrankungen. Arch Exp Veterinarmed (1978) 32:417–25. 211981

[6] Martens EC Neumann M Desai MS. Interactions of commensal and pathogenic microorganisms with the intestinal mucosal barrier. Nat Rev Microbiol (2018) 16:457–70. doi: 10.1038/s41579-018-0036-x 29904082

[7] Olivares-Villagómez D van Kaer L. Intestinal intraepithelial lymphocytes: Sentinels of the mucosal barrier. Trends Immunol (2018) 39:264–75. doi: 10.1016/j.it.2017.11.003 PMC8056148 29221933

[8] Turner JR. Intestinal mucosal barrier function in health and disease. Nat Rev Immunol (2009) 9:799–809. doi: 10.1038/nri2653 19855405

[9] Cortez V Meliopoulos VA Karlsson EA Hargest V Johnson C Schultz-Cherry S. Astrovirus biology and pathogenesis. Annu Rev Virol (2017) 4:327–48. doi: 10.1146/annurev-virology-101416-041742 28715976

[10] Kar Mahapatra S Bhattacharjee S Chakraborty SP Majumdar S Roy S. Alteration of immune functions and Th1/Th2 cytokine balance in nicotine-induced murine macrophages: immunomodulatory role of eugenol and n-acetylcysteine. Int Immunopharmacol (2011) 11:485–95. doi: 10.1016/j.intimp.2010.12.020 21237301

[11] Fujisawa S Murakami Y. Eugenol and its role in chronic diseases. Adv Exp Med Biol (2016) 929:45–66. doi: 10.1007/978-3-319-41342-6_3 27771920

[12] Nisar MF Khadim M Rafiq M Chen J Yang Y Wan CC. Pharmacological properties and health benefits of eugenol: A comprehensive review. Oxid Med Cell Longev (2021) 2021:2497354. doi: 10.1155/2021/2497354 34394824 PMC8357497

[13] Pramod K Ansari SH Ali J. Eugenol: a natural compound with versatile pharmacological actions. Nat Prod Commun (2010) 5:1999–2006. 21299140

[14] Ulanowska M Olas B. Biological properties and prospects for the application of eugenol-a review. Int J Mol Sci (2021) 22(7):3671. doi: 10.3390/ijms22073671 33916044 PMC8036490

[15] Hui Q Ammeter E Liu S Yang R Lu P Lahaye L. Eugenol attenuates inflammatory response and enhances barrier function during lipopolysaccharide-induced inflammation in the porcine intestinal epithelial cells. J Anim Sci (2020) 98(8):skaa245. doi: 10.1093/jas/skaa245 32735667 PMC7531220

[16] Lane T Anantpadma M Freundlich JS Davey RA Madrid PB Ekins S. The natural product eugenol is an inhibitor of the Ebola virus *In vitro* . Pharm Res (2019) 36:104. doi: 10.1007/s11095-019-2629-0 31101988 PMC6668022

[17] Aboubakr HA Nauertz A Luong NT Agrawal S El-Sohaimy SA Youssef MM. *In vitro* antiviral activity of clove and ginger aqueous extracts against feline calicivirus, a surrogate for human norovirus. J Food Prot (2016) 79:1001–12. doi: 10.4315/0362-028X.JFP-15-593 27296605

[18] Anka AU Tahir MI Abubakar SD Alsabbagh M Zian Z Hamedifar H. Coronavirus disease 2019 (COVID-19): An overview of the immunopathology, serological diagnosis and management. Scand J Immunol (2021) 93:e12998. doi: 10.1111/sji.12998 33190302 PMC7744910

[19] Wullaert A Bonnet MC Pasparakis M. NF-κB in the regulation of epithelial homeostasis and inflammation. Cell Res (2011) 21:146–58. doi: 10.1038/cr.2010.175 PMC3193399 21151201

[20] Peuhkuri K Vapaatalo H Korpela R. Even low-grade inflammation impacts on small intestinal function. World J Gastroenterol (2010) 16:1057–62. doi: 10.3748/wjg.v16.i9.1057 PMC2835780 20205274

[21] Hu C Niu X Chen S Wen J Bao M Mohyuddin SG. A comprehensive analysis of the colonic flora diversity, short chain fatty acid metabolism, transcripts, and biochemical indexes in heat-stressed pigs. Front Immunol (2021) 12:717723. doi: 10.3389/fimmu.2021.717723 34745096 PMC8567839

[22] Li J Zhang L Wu T Li Y Zhou X Ruan Z. Indole-3-propionic acid improved the intestinal barrier by enhancing epithelial barrier and mucus barrier. J Agric Food Chem (2021) 69:1487–95. doi: 10.1021/acs.jafc.0c05205 33356219

[23] Gresse R Chaucheyras-Durand F Fleury MA van de Wiele T Forano E Blanquet-Diot S. Gut microbiota dysbiosis in postweaning piglets: Understanding the keys to health. Trends Microbiol (2017) 25:851–73. doi: 10.1016/j.tim.2017.05.004 28602521

[24] Jayaraman B Nyachoti CM. Husbandry practices and gut health outcomes in weaned piglets: A review. Anim Nutr (2017) 3:205–11. doi: 10.1016/j.aninu.2017.06.002 PMC5941228 29767154

[25] Ding H Cao A Li H Zhao Y Feng J. Effects of eucommia ulmoides leaf extracts on growth performance, antioxidant capacity and intestinal function in weaned piglets. J Anim Physiol Anim Nutr (Berl) (2020) 104:1169–77. doi: 10.1111/jpn.13333 32153077

[26] Pirgozliev V Mansbridge SC Rose SP Lillehoj HS Bravo D. Immune modulation, growth performance, and nutrient retention in broiler chickens fed a blend of phytogenic feed additives. Poult Sci (2019) 98:3443–9. doi: 10.3382/ps/pey472 30325468

[27] Poli CH Thornton-Kurth KJ Legako JF Bremm C Hampel VS Hall J. The effect of plant bioactive compounds on lamb performance, intake, gastrointestinal parasite burdens, and lipid peroxidation in muscle. J Anim Sci (2021) 99(1):skab009. doi: 10.1093/jas/skab009 33454733 PMC7851892

[28] Su G Zhou X Wang Y Chen D Chen G Li Y. Dietary supplementation of plant essential oil improves growth performance, intestinal morphology and health in weaned pigs. J Anim Physiol Anim Nutr (Berl) (2020) 104:579–89. doi: 10.1111/jpn.13271 31854008

[29] Su G Zhou X Wang Y Chen D Chen G Li Y. Effects of plant essential oil supplementation on growth performance, immune function and antioxidant activities in weaned pigs. Lipids Health Dis (2018) 17:139. doi: 10.1186/s12944-018-0788-3 29903022 PMC6003089

[30] MArcin AS ed. The effects of aromatic oils on growth performance and physiological parameters in the intestine of weaned pigs. Carol Stream, Ill: Allured Publ. Corp (2006). 5 p.

[31] Luo L Wang S Zhu L Fan B Liu T Wang L. Aminopeptidase n-null neonatal piglets are protected from transmissible gastroenteritis virus but not porcine epidemic diarrhea virus. Sci Rep (2019) 9:13186. doi: 10.1038/s41598-019-49838-y 31515498 PMC6742759

[32] Liang X Wang P Lian K Han F Tang Y Zhang S. APB-13 improves the adverse outcomes caused by TGEV infection by correcting the intestinal microbial disorders in piglets. J Anim Physiol Anim Nutr (Berl) (2022) 106:69–77. doi: 10.1111/jpn.13555 34075636

[33] Pu J Chen D Tian G He J Huang Z Zheng P. All-trans retinoic acid attenuates transmissible gastroenteritis virus-induced inflammation in IPEC-J2 cells *via* suppressing the RLRs/NF-κB signaling pathway. Front Immunol (2022) 13:734171. doi: 10.3389/fimmu.2022.734171 35173714 PMC8841732

[34] Kumar A Siddiqi NJ Alrashood ST Khan HA Dubey A Sharma B. Protective effect of eugenol on hepatic inflammation and oxidative stress induced by cadmium in male rats. BioMed Pharmacother (2021) 139:111588. doi: 10.1016/j.biopha.2021.111588 33862491

[35] Zohar T Loos C Fischinger S Atyeo C Wang C Slein MD. Compromised humoral functional evolution tracks with SARS-CoV-2 mortality. Cell (2020) 183:1508–19.e12. doi: 10.1016/j.cell.2020.10.052 33207184 PMC7608014

[36] Schroeder HW Cavacini L. Structure and function of immunoglobulins. J Allergy Clin Immunol (2010) 125:S41–52. doi: 10.1016/j.jaci.2009.09.046 PMC3670108 20176268

[37] Panda S Ding JL. Natural antibodies bridge innate and adaptive immunity. J Immunol (2015) 194:13–20. doi: 10.4049/jimmunol.1400844 25527792

[38] Hand TW Reboldi A. Production and function of immunoglobulin a. Annu Rev Immunol (2021) 39:695–718. doi: 10.1146/annurev-immunol-102119-074236 33646857

[39] Stacey HD Golubeva D Posca A Ang JC Novakowski KE Zahoor MA. IgA potentiates NETosis in response to viral infection. Proc Natl Acad Sci USA (2021) 118(27):e2101497118. doi: 10.1073/pnas.2101497118 34183391 PMC8271757

[40] Nydegger UE Fierz W Risch L. Benefits and risks of IgA in immunoglobulin preparations. Transfus Apher Sci (2012) 46:97–102. doi: 10.1016/j.transci.2011.11.014 22209283

[41] Buckley A Turner JR. Cell biology of tight junction barrier regulation and mucosal disease. Cold Spring Harb Perspect Biol (2018) 10(1):a029314. doi: 10.1101/cshperspect.a029314 28507021 PMC5749156

[42] Pluske JR Thompson MJ Atwood CS Bird PH Williams IH Hartmann PE. Maintenance of villus height and crypt depth, and enhancement of disaccharide digestion and monosaccharide absorption, in piglets fed on cows’ whole milk after weaning. Br J Nutr (1996) 76:409–22. doi: 10.1079/bjn19960046 8881713

[43] Escudero-Hernández C. Epithelial cell dysfunction in coeliac disease. Int Rev Cell Mol Biol (2021) 358:133–64. doi: 10.1016/bs.ircmb.2020.09.007 33707053

[44] Meng Y Zhang Y Liu M Huang YK Zhang J Yao Q. Evaluating intestinal permeability by measuring plasma endotoxin and diamine oxidase in children with acute lymphoblastic leukemia treated with high-dose methotrexate. Anticancer Agents Med Chem (2016) 16:387–92. doi: 10.2174/1871520615666150812125955 26265099

[45] Pohanka M. D-lactic acid as a metabolite: Toxicology, diagnosis, and detection. BioMed Res Int (2020) 2020:3419034. doi: 10.1155/2020/3419034 32685468 PMC7320276

[46] Garcia MA Nelson WJ Chavez N. Cell-cell junctions organize structural and signaling networks. Cold Spring Harb Perspect Biol (2018) 10(4):a029181. doi: 10.1101/cshperspect.a029181 28600395 PMC5773398

[47] Pu J Chen D Tian G He J Huang Z Zheng P. All-trans retinoic acid attenuates transmissible gastroenteritis virus-induced apoptosis in IPEC-J2 cells *via* inhibiting ROS-mediated P38MAPK signaling pathway. Antioxidants (Basel) (2022) 11(2):345. doi: 10.3390/antiox11020345 35204227 PMC8868330

[48] Zhao S Gao J Zhu L Yang Q. Transmissible gastroenteritis virus and porcine epidemic diarrhoea virus infection induces dramatic changes in the tight junctions and microfilaments of polarized IPEC-J2 cells. Virus Res (2014) 192:34–45. doi: 10.1016/j.virusres.2014.08.014 25173696 PMC7114495

[49] Huang G Zhao Di Lan C Wu B Li X Lou S. Glucose-assisted trophic conversion of chlamydomonas reinhardtii by expression of glucose transporter GLUT1. Algal Res (2022) 62:102626. doi: 10.1016/j.algal.2021.102626

[50] Nishimura K Fujita Y Ida S Yanagimachi T Ohashi N Nishi K. Glycaemia and body weight are regulated by sodium-glucose cotransporter 1 (SGLT1) expression *via* O-GlcNAcylation in the intestine. Mol Metab (2022) 59:101458. doi: 10.1016/j.molmet.2022.101458 35189429 PMC8902621

[51] Shu R Wang C Meng Q Liu Z Wu J Sun P. Resveratrol enhances the protective effects of JBP485 against indomethacin-induced rat intestinal damage *in vivo* and vitro through up-regulating oligopeptide transporter 1 (Pept1). BioMed Pharmacother (2019) 111:251–61. doi: 10.1016/j.biopha.2018.12.084 30590313

[52] Ingersoll SA Ayyadurai S Charania MA Laroui H Yan Y Merlin D. The role and pathophysiological relevance of membrane transporter PepT1 in intestinal inflammation and inflammatory bowel disease. Am J Physiol Gastrointest Liver Physiol (2012) 302:G484–92. doi: 10.1152/ajpgi.00477.2011 PMC3311434 22194420

[53] Shima Y Maeda T Aizawa S Tsuboi I Kobayashi D Kato R. L-arginine import *via* cationic amino acid transporter CAT1 is essential for both differentiation and proliferation of erythrocytes. Blood (2006) 107:1352–6. doi: 10.1182/blood-2005-08-3166 16210335

[54] Strobel J Mieth M Endress B Auge D König J Fromm MF. Interaction of the cardiovascular risk marker asymmetric dimethylarginine (ADMA) with the human cationic amino acid transporter 1 (CAT1). J Mol Cell Cardiol (2012) 53:392–400. doi: 10.1016/j.yjmcc.2012.06.002 22705145

[55] Torres J Mehandru S Colombel J-F Peyrin-Biroulet L. Crohn’s disease. Lancet (2017) 389:1741–55. doi: 10.1016/S0140-6736(16)31711-1 27914655

[56] Ungaro R Mehandru S Allen PB Peyrin-Biroulet L Colombel J-F. Ulcerative colitis. Lancet (2017) 389:1756–70. doi: 10.1016/S0140-6736(16)32126-2 PMC6487890 27914657

[57] Fasano A. Zonulin and its regulation of intestinal barrier function: the biological door to inflammation, autoimmunity, and cancer. Physiol Rev (2011) 91:151–75. doi: 10.1152/physrev.00003.2008 21248165

[58] Luo C Huang C Zhu L Kong L Yuan Z Wen L. Betulinic acid ameliorates the T-2 toxin-triggered intestinal impairment in mice by inhibiting inflammation and mucosal barrier dysfunction through the NF-κB signaling pathway. Toxins (Basel) (2020) 12(12):794. doi: 10.3390/toxins12120794 PMC7763746 33322178

[59] Zhai Z Wang J Huang B Yin S. Low-fat yogurt alleviates the pro-inflammatory cytokine IL-1β-induced intestinal epithelial barrier dysfunction. J Dairy Sci (2019) 102:976–84. doi: 10.3168/jds.2018-15226 30580944

[60] Chen S Liu H Li Z Tang J Huang B Zhi F. Epithelial PBLD attenuates intestinal inflammatory response and improves intestinal barrier function by inhibiting NF-κB signaling. Cell Death Dis (2021) 12:563. doi: 10.1038/s41419-021-03843-0 34059646 PMC8166876

