# Supplementary material for: Eugenol alleviates transmissible gastroenteritis virus-induced intestinal epithelial injury by regulating NF-κB signaling pathway
Source: Front Immunol. 2022 Aug 16;13:921613. doi: 10.3389/fimmu.2022.921613 (PMC9427193; doi:10.3389/fimmu.2022.921613)
Supplement: Supplementary file 1 [file Table_1.docx]

Table S1. Primer sequences table

| Gene | Primers | Sequences | Product size | Accession numbers |
| --- | --- | --- | --- | --- |
| β-actin | Forward | GCAAATGCTTCTAGGCGGAC | 148 | XM_021086047.1 |
|  | Reverse | GCGTCCATCACAGCTTCTCA |  |  |
| NF-κB | Forward | GGGGCGATGAGATCTTCCTG | 110 | NM_001114281.1 |
|  | Reverse | CACGTCGGCTTGTGAAAAGG |  |  |
| IL-6 | Forward | GCTGCAGTCACAGAACGAGT | 118 | NM_214399.1 |
|  | Reverse | CAGGTGCCCCAGCTACATTA |  |  |
| IL-10 | Forward | GACCAGATGGGCGACTTGTT | 120 | NM_214041.1 |
|  | Reverse | TGCCTTCGGCATTACGTCTT |  |  |
| GluT-1 | Forward | GCCGGACCTTCGATGAGATT | 71 | XM_021096908.1 |
|  | Reverse | CTCGGGTGTCTTGTCGCTTT |  |  |
| GluT-2 | Forward | TGGGCTAATTTCAGGCTTGGT | 79 | NM_001097417.1 |
|  | Reverse | AAGAGCACCAATAGCACCCC |  |  |
| ZnT-1 | Forward | ACGCTACCACCATTCAACCT | 112 | NM_001139470.1 |
|  | Reverse | CGTGTCCCACAACATTGCTT |  |  |
| PepT-1 | Forward | CAGACTTCGACCACAACGGA | 99 | NM_214347.1 |
|  | Reverse | TTATCCCGCCAGTACCCAGA |  |  |
| SglT1 | Forward | AGCTACCTCAAGATGCTGCC | 75 | NM_001012297.1 |
|  | Reverse | ATCATCTGGAAACAGCACGC |  |  |
| CaT1 | Forward | GCCTGAACAATGCCACGAAG | 109 | XM_021065165.1 |
|  | Reverse | CCCACGAAGGCATAGAAGCA |  |  |
