# Supplementary figures and images for: Eugenol alleviates transmissible gastroenteritis virus-induced intestinal epithelial injury by regulating NF-κB signaling pathway
Source: Front Immunol. 2022 Aug 16;13:921613. doi: 10.3389/fimmu.2022.921613 (PMC9427193; doi:10.3389/fimmu.2022.921613)

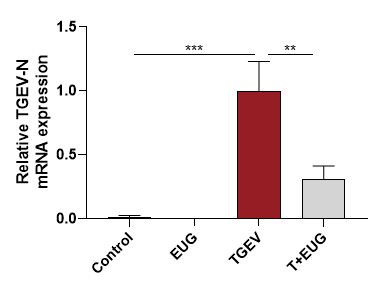

Supplement: Supplementary file 2 [file Image_1.tif]
